# 3D‐Printed Bioadhesive Interfaces as Proliferative Niches for Tissue Repair

**DOI:** 10.1111/cpr.70286

**Published:** 2026-10-06

**Authors:** Zhen Gu, Xinyong Su, Guoshi Xu, Yongqiang Wen, Qi Gu

**Affiliations:** ^1^ School of Chemistry and Biological Engineering, University of Science and Technology Beijing Beijing China; ^2^ Human Organ Physiopathology Emulation System, State Key Laboratory of Organ Regeneration and Reconstruction, Institute of Zoology, Chinese Academy of Sciences Beijing China; ^3^ Beijing Institute for Stem Cell and Regenerative Medicine Beijing China; ^4^ University of Chinese Academy of Sciences Beijing China

**Keywords:** 3D printing, bioadhesives, cell proliferation, interfacial microenvironment, proliferative niche, tissue repair

## Abstract

Bioadhesives have traditionally been viewed as materials for wound closure, sealing and haemostasis However, in tissue repair they also create the first engineered interface that influences cell retention, microenvironmental homeostasis and subsequent remodelling Recent advances in 3D printing are shifting bioadhesives from passive, uniform layers to architecturally controlled interfaces with spatially programmable chemistry, mechanics, transport and biological function. In this Review, we propose an interface‐centred framework in which qualifying 3D‐printed bioadhesive interfaces function as proliferative niches. Such interfaces require stable host‐tissue adhesion and a biocompatible, cell‐permissive microenvironment. They should also measurably support cell survival, context‐appropriate proliferation and constructive remodelling in defined target‐cell populations. We summarize the key design principles that govern proliferative‐niche formation, with printability‐aware fabrication treated as a cross‐cutting prerequisite for implementing interfacial adhesion, mechanical matching, mass transport, surface chemistry and temporal evolution. We then discuss the mechanistic basis through which printed bioadhesive interfaces regulate early protein adsorption, immune activation, mechanotransduction, metabolic exchange and long‐term remodelling trajectories. Representative application contexts, including sealing and haemostasis musculoskeletal repair, wearable and implantable biointerfaces and organoid‐related in vitro models, are examined to highlight application‐specific opportunities and trade‐offs. We further outline emerging evaluation and modelling frameworks that move beyond adhesion strength towards multidimensional assessment, cross‐scale structure–property–function mapping and data‐driven predictive design. By reframing bioadhesives as active interfacial regulators rather than passive fixation materials, this Review provides a conceptual framework for engineering 3D‐printed bioadhesives that not only adhere and seal, but also actively guide constructive tissue repair.

## Graphical abstract

3D printing transforms bioadhesives into structured, multifunctional interfaces that coordinate adhesion, mechanics, transport and biofunctional signalling and may thereby enable proliferative‐niche formation. Qualifying interfaces support cell survival, context‐appropriate proliferation and constructive remodelling advancing tissue repair towards adaptive, predictive and translational bioadhesive design.

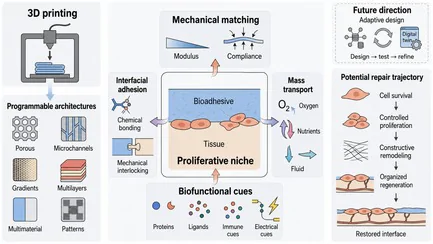

## 1 Introduction: From Bioadhesion to 3D‐Printed Proliferative Niches

Bioadhesives have traditionally been viewed as materials for wound closure, sealing and haemostasis, but in tissue repair their role is broader and more consequential. Rather than serving as passive fixation layers, bioadhesives create the first engineered interface that stabilizes injured tissue and shapes the local conditions for subsequent repair [1, 2]. Conventional evaluation has mainly emphasized adhesion strength, sealing performance and hemostatic efficiency, yet this framework often underestimates how adhesive interfaces regulate cell retention, microenvironmental homeostasis and later remodelling [3, 4]. This limitation is evident in established clinical adhesive classes such as cyanoacrylates, fibrin glues and PEG‐based sealants, which may achieve closure but still show important trade‐offs in compliance, durability, swelling or intrinsic bioactivity depending on the repair scenario [3, 5]. Bioadhesives should therefore be understood not simply as closure materials, but as active interfacial regulators whose biological and structural performance can influence whether repair proceeds towards regeneration or dysfunction [1, 4, 6].

3D printing is significant in this context not merely as a manufacturing method, but because it transforms bioadhesives from uniform layers into architecturally controlled interfaces with spatially programmable chemistry, mechanics, transport and biological function [6, 7]. Compared with conventional coatings, glues or cast patches, printed bioadhesives can integrate geometry control, patient‐specific conformity, multimaterial organization and tunable crosslinking within a single design framework, thereby allowing interfacial properties to be coordinated rather than optimized in isolation [7, 8]. As summarized in Figure 1, this transition extends from basic bioprinting modes to more advanced fabrication strategies that increase structural complexity, interfacial heterogeneity and functional programmability. Recent examples, including direct‐ink‐write tissue adhesives, adaptive printed hydrogel bioadhesives and light‐driven programmable dopamine‐based systems, further show that printing is no longer just a way to shape adhesive materials, but a route to engineer structured microenvironments for repair [6, 8, 9].

**FIGURE 1:**
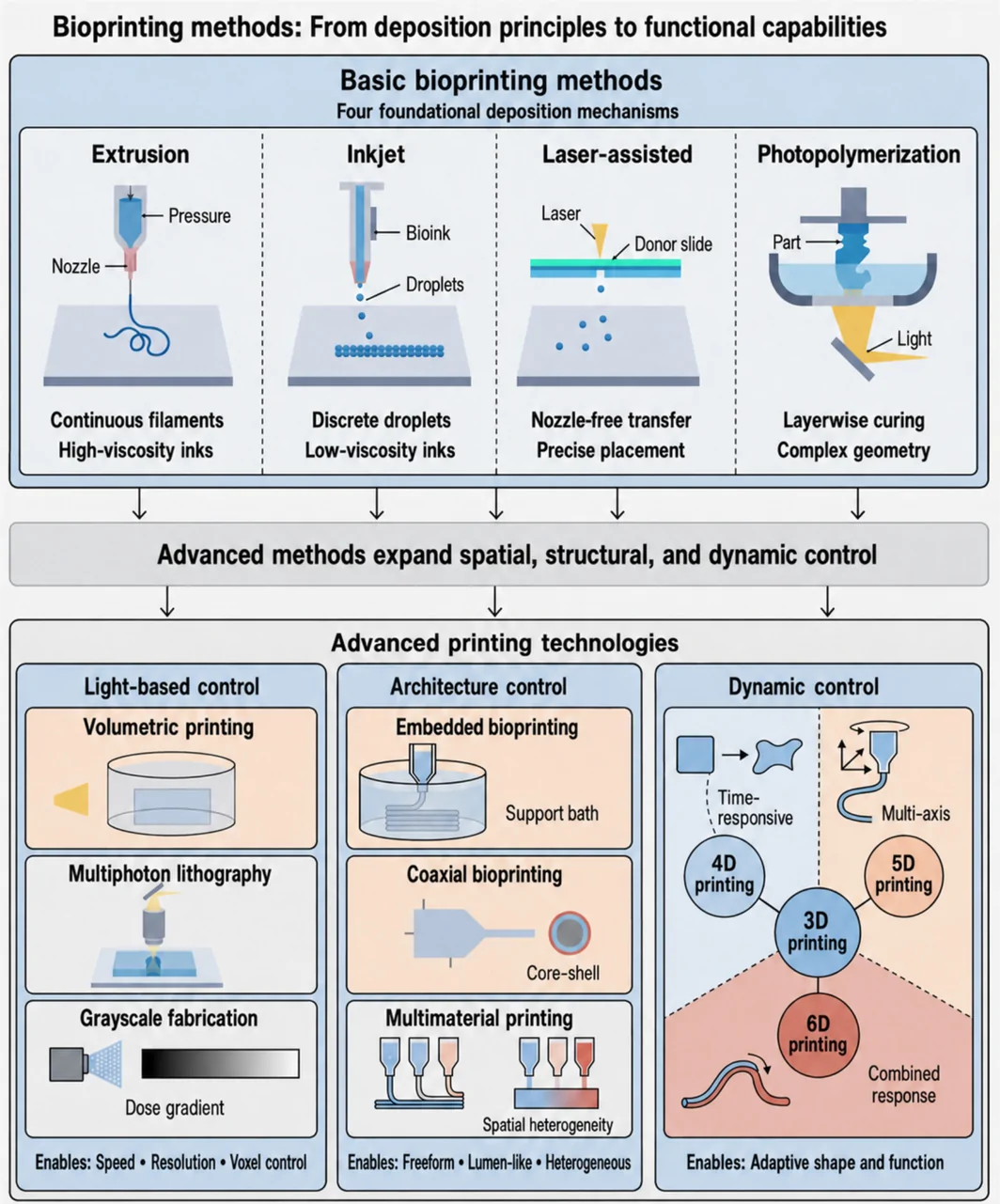
Bioprinting strategies that transform bioadhesives from passive layers into architecturally controlled interfaces. Illustration of the conceptual transition from basic bioprinting methods, including extrusion, droplet/inkjet, laser‐assisted printing and selective photopolymerization, to advanced fabrication technologies such as volumetric printing, multiphoton lithography, grayscale fabrication, embedded printing, coaxial bioprinting, multimaterial printing and multidimensional printing. Together, these approaches expand bioadhesive design from simple deposition towards greater spatial control, structural complexity, interfacial heterogeneity and adaptive functionality.

In this Review, we define a proliferative niche as a deliberately engineered bioadhesive–tissue interface that supports cell survival, context‐appropriate proliferation and constructive remodelling while maintaining stable integration with host tissue [10, 11]. The term is not intended to denote a new material class or an equivalent of the native in vivo stem cell niche. Instead, it provides an interface‐centred framework that links printing‐defined architecture and bioadhesion to measurable biological outcomes. The distinctive contribution of 3D printing lies in its spatial programmability, multimaterial heterogeneity and application‐specific geometry, which are difficult to achieve with conventional bulk bioadhesives [6, 8]. Accordingly, not every 3D‐printed bioadhesive interface constitutes a proliferative niche. Qualification requires stable tissue adhesion over the application‐relevant period, a biocompatible and cell‐permissive interfacial environment and measurable evidence of context‐appropriate proliferation and, where relevant, constructive remodelling in a defined target‐cell population. Depending on the repair setting, the intended targets may be resident, recruited or transplanted cells. Proliferation is treated as a tissue‐ and phase‐appropriate response rather than an outcome to be maximized; persistent or dysregulated expansion associated with pathological remodelling falls outside this framework. Immune modulation, mechanical guidance and temporal adaptability are application‐dependent means of achieving these criteria rather than universal defining requirements. As outlined in Figure 2, we organize this Review around 3D‐printing‐enabled bioadhesive architectures and the key determinants of proliferative‐niche formation, including adhesion, mechanics, transport and biofunctional signalling. We then connect these design factors to downstream repair outcomes and emerging approaches in evaluation, modelling and predictive design. In this way, the translational intent of this Review is to position 3D‐printed bioadhesives not as isolated material innovations, but as an emerging platform for engineering clinically actionable proliferative niches in tissue repair [12, 13].

**FIGURE 2:**
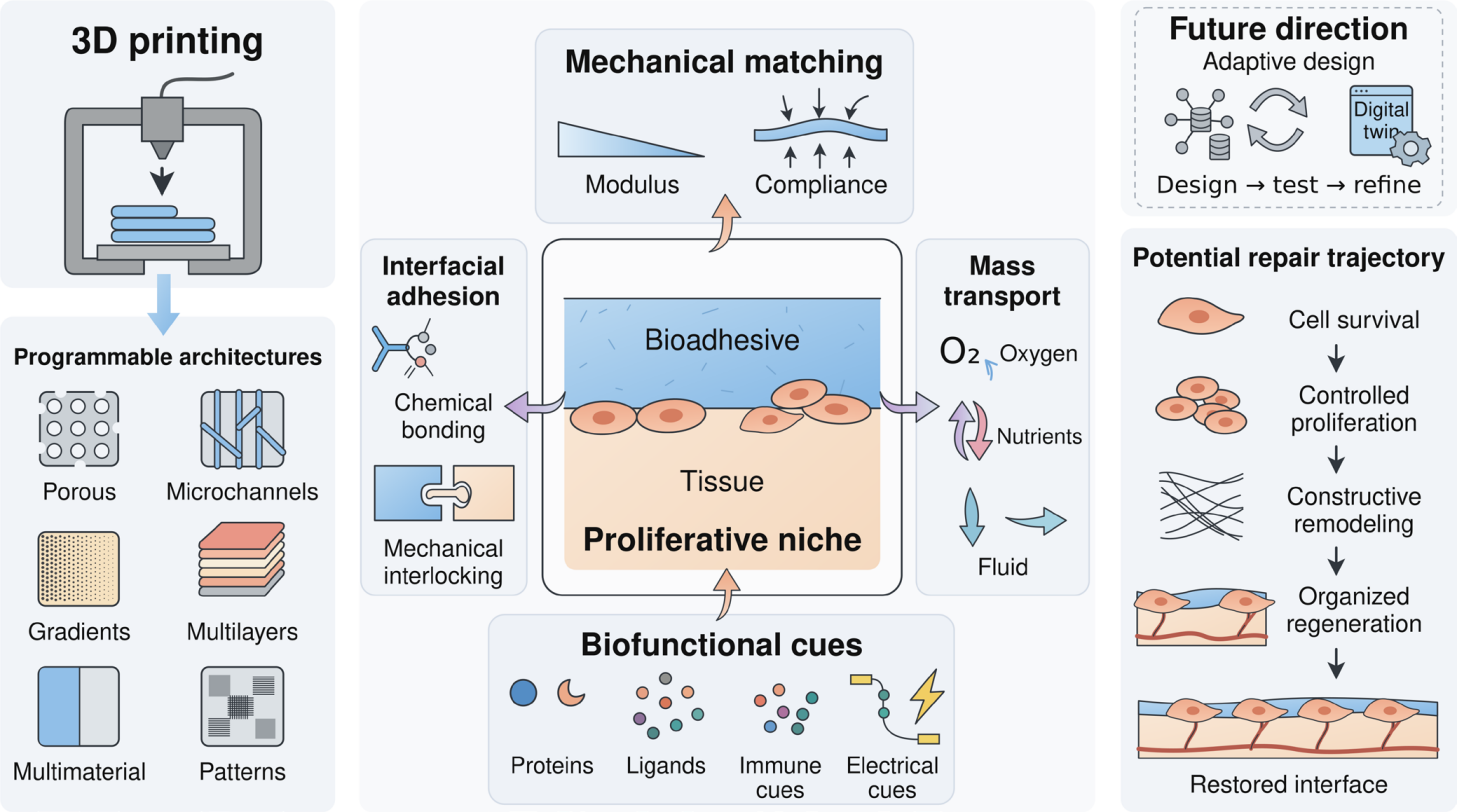
3D printing‐enabled bioadhesive architectures regulate proliferative‐niche formation and tissue‐repair outcomes. Representative printed architectures shape the bioadhesive‐tissue interface through adhesion, mechanics, transport and biofunctional signalling, leading to improved integration, reduced fibrosis, enhanced cell survival and context‐appropriate proliferation, constructive remodelling and organized tissue regeneration, with future progression towards adaptive and predictive design.

## 2 Design Principles for Bioadhesives Through a Proliferative‐Niche Lens

A proliferative‐niche‐centred framework is essential for the design of 3D‐printed bioadhesives. Here, printability is treated as a cross‐cutting manufacturing prerequisite rather than a downstream biological property because it defines which combinations of interfacial chemistry, network mechanics, transport architecture and temporal behaviour can be fabricated reproducibly. Sections 2.1, 2.5 first define the intended interfacial and biological functions, whereas Section 2.6 then integrates the fabrication constraints that enable and delimit their implementation. Throughout this chapter, directly measured process–property and structure–function relationships are distinguished from proposed biological implications and numerical values are presented as study‐specific examples rather than universal design thresholds. Rather than considering adhesion, mechanics, transport, surface chemistry, temporal behaviour and printability as separate material properties, these parameters should be understood as acting together to determine whether the bioadhesive–tissue interface can support stable contact, microenvironmental homeostasis and constructive remodelling. In this view, interfacial design is not merely a question of bonding or structural fixation, but of engineering a cell‐permissive interface in which physical, chemical and architectural factors are coordinated to guide proliferation and repair. As summarized in Figure 3, these design variables are interdependent and their integration ultimately governs the transition from engineering input to interfacial proliferative‐niche state and downstream proliferative outcomes.

**FIGURE 3:**
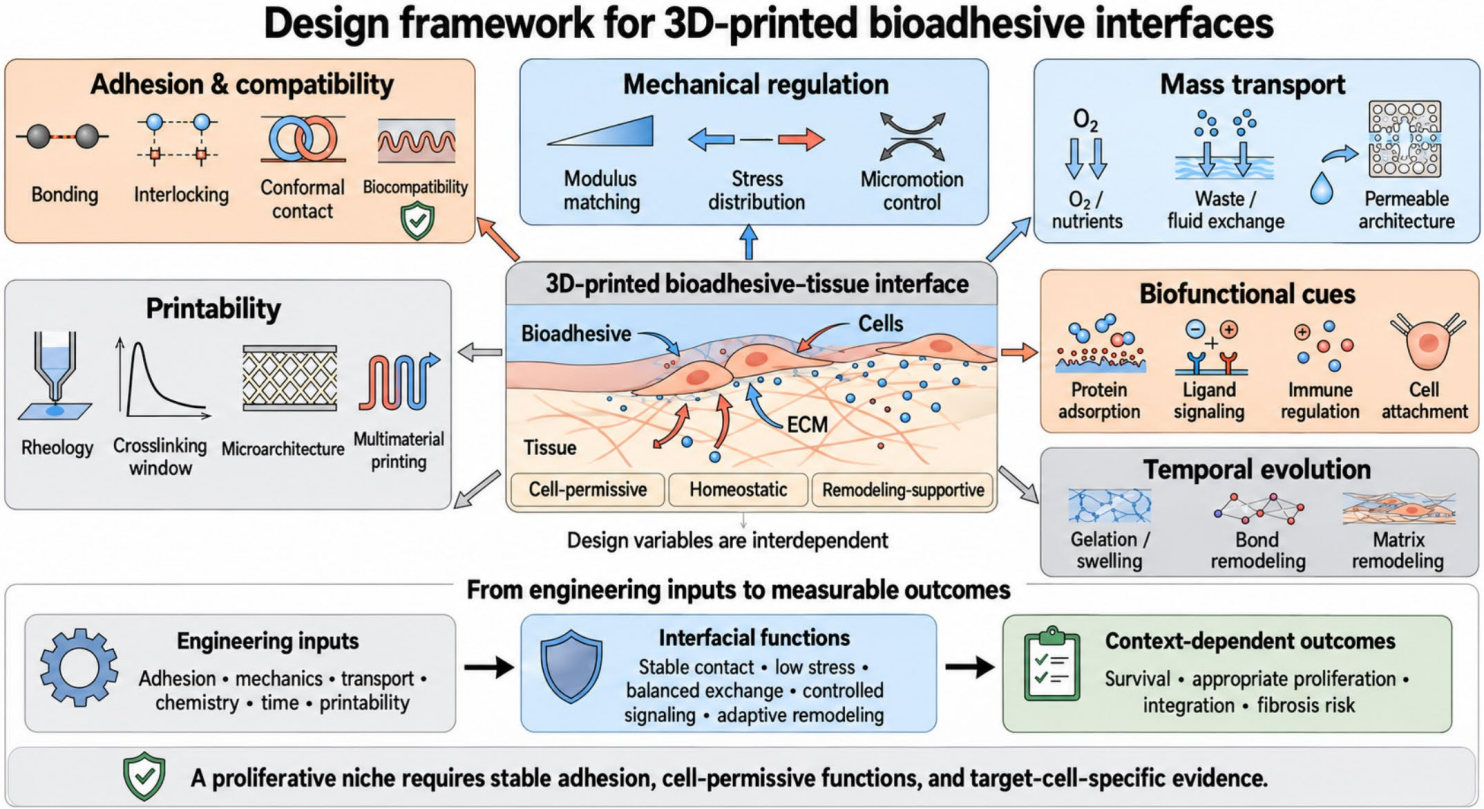
Design principles of 3D‐printed bioadhesives through a proliferative‐niche lens. Printability is presented as a cross‐cutting manufacturing prerequisite that defines the feasible processing window for implementing interfacial adhesion, mechanical matching, mass transport, surface chemistry and temporal evolution. The lower matrix organizes these variables as a testable pathway from engineering parameters to interfacial proliferative‐niche states and biological outcomes. The arrows represent reported or proposed relationships whose evidentiary strength depends on whether the processing, interfacial and biological variables were measured within matched experimental designs; they should not be interpreted as universally established causal relationships.

### 2.1 Interfacial Adhesion Mechanisms and Tissue Compatibility

Interfacial adhesion is the foundational design requirement of bioadhesives because it determines not only whether a material can establish stable contact with wet and dynamic tissues but also whether the resulting interface can support a permissive proliferative niche for subsequent repair. In this context, effective bioadhesion arises from the coordinated action of covalent and noncovalent interactions, topological entanglement and physical interlocking, while tissue compatibility depends on maintaining a hydrated, non‐toxic and mechanically stable microenvironment at the interface [1, 7]. Importantly, the balance between crosslinking kinetics and interfacial diffusion governs whether the adhesive can achieve sufficient contact formation before network fixation, which directly influences both adhesion durability and cellular tolerance [7]. Meanwhile, biomimetic and catechol‐containing systems further highlight that interfacial chemistry should be designed not only for wet adhesion but also for cytocompatibility, matrix stability and controlled remodelling so that the adhesive layer functions as an active proliferative niche rather than a passive sealant [14, 15, 16, 17].

### 2.2 Mechanical Matching, Compliance Gradients and Stress Distribution

Mechanical matching is a central design requirement because a proliferative niche cannot be sustained at an interface that is mechanically hostile to cells or unstable under physiological loading. In 3D‐printed bioadhesives, this principle is governed mainly by three coupled parameters. Modulus matching is intended to reduce abrupt load transfer between the adhesive and host tissue. Compliance gradients smooth the transition from soft tissue to stiffer functional or device‐bearing regions. Stress distribution determines whether forces are dissipated broadly or concentrated at failure‐prone sites. When these parameters are rationally tuned, they may reduce micromotion, crack initiation and mechanically induced irritation, thereby creating conditions that are more permissive for cell spreading and remodelling however, these biological effects require direct validation. Conversely, when mismatch persists, even strong initial adhesion may evolve into biological non‐permissiveness. For this reason, mechanics in bioadhesive design should be viewed not simply as a structural consideration, but as a core regulator of the proliferative niche that must next be integrated with transport‐related factors such as hydration, permeability and metabolite exchange [12, 14, 18, 19, 20]. Importantly, the current literature does not establish a universal modulus‐mismatch range that optimizes cell proliferation. In the same PU–PAA–NHS adhesive formulation, increasing the printed fill density from 56% to 89% increased the effective initial Young's modulus from 29.2 ± 5.7 to 148.6 ± 12.7 kPa, corresponding to an approximately 5.1‐fold architectural modulation [6]. This comparison establishes a ‘printing architecture → effective mechanics’ relationship, but not an ‘architecture → proliferation’ relationship, because proliferation was not compared among the printed geometries. In a complementary bioprinted hydrogel model with fixed PECMA and RGD contents, increasing the Ca^2+^ concentration from 6 to 10 mM shortened the stress‐relaxation time from approximately 470 to 248 s. In the faster‐relaxing region, fibroblasts began to spread within 24 h and formed interconnected networks by Day 7, whereas cell viability remained similar [21]. These findings indicate that static modulus alone is insufficient and that time‐dependent mechanics should be linked to target‐cell‐specific outcomes in matched experimental designs.

### 2.3 Mass Transport: Permeability, Hydration and Metabolite Exchange

Mass transport is essential because a proliferative niche requires not only stable adhesion but also a chemically permissive interfacial environment that sustains cell survival and proliferation [1, 7]. In practice, this design factor is governed mainly by permeability, hydration balance and metabolite/fluid handling. Permeability can influence oxygen and nutrient diffusion. Hydration balance determines whether the interface remains conformal without excessive swelling. Metabolite and fluid handling help prevent waste accumulation and long‐term interfacial destabilization, especially in wearable or occlusive systems [6, 22, 23]. Accordingly, transport design can influence cell proliferation and remodelling although a causal claim requires that the printed architecture, transport state and biological outcome be measured within the same experimental system. Quantitative examples from closely matched bioprinted systems illustrate this conditional relationship. In a FLight‐printed hydrogel system containing microchannels with an average diameter of approximately 3.5 μm, about 76% of fibroblasts were Ki67‐positive after 3 days in the 3D matrix, compared with 19% on the corresponding 2D FLight matrix, representing an approximately fourfold difference [24]. In vertical cryo‐bioprinted GelMA constructs, changing the freezing‐plate temperature from −5°C to −20°C tuned the channel size from 184.81 ± 26.19 to 23.80 ± 5.0 μm. Within the same printed filament, cell viability in the frozen, microchannel‐containing region reached 94.01% ± 2.34% on Day 7, compared with 88.90°C ± 1.63% in the unfrozen region [24]. These comparisons provide quantitative ‘architecture → biological outcome’ examples, but oxygen concentration was not directly measured as the mediating variable. The reported dimensions should, therefore, be interpreted as study‐specific examples rather than universal optimal pore‐size thresholds for 3D‐printed bioadhesives. Once an appropriate exchange environment is established, the next question becomes how surface chemistry and signalling presentation further instruct interfacial biological behaviour [10, 25].

### 2.4 Surface Chemistry and Biofunctional Signalling Cues

Overall, surface chemistry is critical because it determines the first biological identity of a 3D‐printed bioadhesive interface and therefore whether that interface becomes a permissive proliferative niche for repair [26, 27]. Specifically, three parameters are central: redox‐active adhesive motifs such as polyphenols and catechols that regulate both wet bonding and ROS balance; charge and asymmetric biochemical presentation that control protein adsorption, cell attachment and immune recognition; and biofunctional signalling cues that promote M2 polarization, angiogenesis, stem/stromal cell recruitment and vascularized regeneration [27, 28, 29, 30, 31]. Taken together, surface chemistry does not simply stabilize contact but programmes the interfacial trajectory towards cell proliferation and constructive remodelling rather than fibrotic isolation, which in turn raises the next question of how these cues are maintained or transformed during gelation, degradation and long‐term interfacial evolution [26, 28].

### 2.5 Temporal Properties: Gelation, Degradation and Interfacial Evolution

Temporal properties are important because a proliferative niche is inherently dynamic: the success of a 3D‐printed bioadhesive depends not only on whether it adheres initially, but on whether its time‐dependent behaviour continues to support cell survival, proliferation and constructive remodelling after application or implantation [4, 7]. In practice, this dimension is governed mainly by three coupled parameters. Gelation and crosslinking kinetics determine early fixation, conformal contact and interfacial diffusion. Degradation and swelling behaviour regulate how long structural and biochemical support is maintained. Interfacial evolution, including bond rearrangement and matrix replacement, influences whether the interface progresses towards regeneration or failure [7, 9, 32, 33]. Taken together, temporal control shapes the full trajectory of cell proliferation and remodelling rather than a single endpoint. These temporal targets must, however, be implemented within a feasible processing window; Section 2.6 therefore considers printability as the cross‐cutting manufacturing constraint that couples rheology and fixation to adhesion, mechanics, transport and degradation [7, 9].

### 2.6 Printability as a Cross‐Cutting Manufacturing Prerequisite: Rheology, Crosslinking Windows and Architectural Freedom

Printability is the manufacturing gate through which the intended interfacial functions described above must be realized. For extrusion‐based systems, the ink must flow through the nozzle under shear, recover sufficient viscosity and elastic character after deposition and be fixed within a time window that preserves both shape and tissue‐contact capability. This requirement does not impose a universal upper limit on the final crosslinking density; it primarily constrains the extent and reversibility of network formation before and during deposition, whereas additional fixation may occur after printing [6, 7, 34, 35, 36].

These stages create coupled, but not universally monotonic, trade‐offs. Increasing polymer concentration or pre‐deposition network formation can improve yield behaviour and shape retention, but may also increase extrusion pressure or promote clogging. Premature network fixation may further restrict the chain mobility and conformal wetting required for interfacial contact, whereas delayed fixation can preserve conformability but permit spreading or weak early cohesion [6, 7]. In one direct‐ink‐write tissue adhesive, total polymer concentrations below 15 wt% led to spreading, whereas concentrations above 25 wt% were difficult to extrude because of nozzle clogging. Within the printable window, printing parameters and fill geometry were subsequently used to tune filament and pore dimensions, effective modulus and nonlinear mechanical responses [6].

Post‐deposition crosslinking provides an additional control level. Stronger or faster fixation can improve dimensional stability and cohesion but, depending on the network chemistry, may increase stiffness, reduce stress relaxation and molecular transport and slow degradation. These changes may narrow the ranges available for mechanical matching and mass exchange, although their magnitude and biological consequences remain formulation‐ and tissue‐specific [7, 18, 34]. Printed meshes, channels, gradients and multimaterial patterns can partially decouple these constraints by tuning effective compliance, fluid handling and transport at the architectural level without necessarily changing the bulk formulation, although open geometries must be balanced against continuous load‐bearing and adhesive contact area [6, 34].

The reported concentration and architectural effects establish process–structure and architecture–mechanics relationships, but they do not by themselves demonstrate that a particular printing condition or geometry improves cell proliferation. Establishing that causal step requires matched‐composition studies in which one processing or architectural parameter is varied while interfacial conditions and quantitative, target‐cell‐specific biological outcomes are measured. Printability should therefore be optimized as a processing window linking pre‐print rheology, post‐print network formation and architecture, with biological claims calibrated to the corresponding level of direct evidence.

## 3 Mechanistic Basis: How Bioadhesive Interfaces Regulate Cell Proliferation and Remodelling

The regenerative function of 3D‐printed bioadhesives depends not only on adhesion itself, but on how the resulting interface regulates protein adsorption, immune activation, mechanotransduction, transport, signalling and temporal remodelling Together, these processes determine whether the bioadhesive–tissue interface develops into a proliferative niche that supports constructive repair. The following sections therefore focus on the mechanistic basis through which interfacial design is converted into specific patterns of cell proliferation and tissue remodelling.

### 3.1 Early Interfacial Events: Protein Adsorption, Immune Activation and Microenvironment Priming

Early interfacial events occur at the very beginning of the healing timeline and they prime later remodelling trajectories by determining the first biological identity of the bioadhesive–tissue interface [26]. Mechanistically, this stage is dominated by protein adsorption, innate immune activation, ROS balance and early cytokine dynamics, which together shape whether cells encounter a pro‐healing or pro‐fibrotic microenvironment [29, 37, 38]. These responses are in turn regulated by material chemistry, redox‐active motifs, wet‐contact stability, swelling behaviour Janus or asymmetric architectures and printing‐enabled control over local surface presentation and transport [10, 39, 40]. Therefore, the engineering implication is clear: bioadhesive design should first optimize the early immune and adsorption landscape of the interface, because this initial priming largely determines whether subsequent proliferation and tissue remodelling proceed towards regeneration or dysfunction [26, 29].

### 3.2 Mechanotransduction and Cell‐Cycle Regulation at Adhesive Interfaces

Mechanotransduction takes place after the earliest contact and priming events, when cells begin to translate the physical properties of the adhesive interface into biological decisions about spreading, proliferation and phenotype stability [19]. Mechanistically, this stage is governed by integrin engagement, focal adhesion maturation, cytoskeletal tension and downstream pathways such as FAK, RhoA/ROCK, YAP/TAZ, Piezo1 and β‐catenin, which together connect interfacial mechanics to cell‐cycle regulation and tissue‐specific gene expression [21, 41]. These responses are in turn shaped by stiffness, stress relaxation, crosslinking mode, microtopography and printed anisotropic architectures, all of which determine how forces are sensed, dissipated and spatially organized at the interface [14, 42]. Therefore, the engineering implication is that bioadhesive design should optimize mechanically instructive, not merely mechanically stable, interfaces, because this is a major determinant of whether proliferation proceeds towards functional regeneration or mechanically driven dysfunction [18, 19].

### 3.3 Transport‐Coupled Regulation of Proliferation: Oxygen, Metabolites and Fluid Handling

Transport‐coupled regulation acts after the earliest interfacial attachment and priming stages, when sustained proliferation becomes dependent on whether the proliferative niche at the bioadhesive interface can maintain oxygenation, nutrient supply, pH stability and waste clearance over time [6, 22]. Mechanistically, this stage is governed by diffusion of oxygen and nutrients, removal of metabolites and excess exudate, management of interfacial water films and control of redox balance, all of which determine whether cells remain viable, proliferative and repair‐competent or instead shift towards oxidative stress and dysfunction [28, 43, 44]. These responses are in turn regulated by permeability, swelling, pore or channel architecture, hydrophilic/hydrophobic balance and printing‐enabled control over mesh geometry, drainage and local fluid exchange; in wearable systems, sweat handling also becomes part of the same transport logic because it affects both tissue homeostasis and signal fidelity [6, 23]. Therefore, the engineering implication is that bioadhesive design should optimize transport‐supportive and fluid‐stable interfaces, because a proliferative niche that cannot exchange effectively will not sustain constructive proliferation even if it adheres well initially [22, 23].

### 3.4 Bioelectrical and Biochemical Crosstalk in Multifunctional Interfaces

Bioelectrical and biochemical crosstalk emerges when the bioadhesive interface begins to act as an active communication layer rather than a passive seal, especially in neural, cardiac and wearable bioelectronic settings [20, 45]. Mechanistically, this stage is governed by the coupling of electrical sensing or stimulation with biochemical regulation such as anti‐inflammatory signalling drug release or regenerative cue presentation, meaning that proliferation and remodelling are affected by both electrophysiological stability and molecular microenvironment control [46, 47]. These responses are in turn shaped by conductive hydrogel chemistry, mixed ionic‐electronic transport, tissue‐like mechanics, robust adhesive integration and printing‐enabled spatial organization of conductive, adhesive and therapeutic domains [48, 49, 50]. Therefore, the engineering implication is that multifunctional bioadhesive interfaces should be designed as proliferative niches with coupled electro‐biochemical functions, because only interfaces that simultaneously preserve communication fidelity and biological compatibility are likely to support long‐term constructive remodelling [20, 45].

### 3.5 Temporal Remodelling Trajectories: From Acute Integration to Chronic Function

Temporal remodelling trajectories occupy the latest stage of the healing sequence, because the real performance of a proliferative niche is revealed across acute integration, subacute adaptation and chronic function rather than at a single early endpoint [32]. Mechanistically, this stage is governed by time‐dependent shifts in immune resolution, cell infiltration, angiogenesis, ECM deposition, load transfer and tissue‐specific maturation, which together determine whether the interface proceeds towards regeneration or chronic dysfunction [26, 51]. These outcomes are in turn regulated by material bioadaptability, degradation timing, dynamic bond behaviour stress‐redistributing architectures and printing‐enabled control over regional structure and function; in some systems, they can also be monitored through imaging or preserved through stable bioelectronic interfacing over weeks [20, 52]. Therefore, the engineering implication is that bioadhesive design should optimize phase‐matched evolution of the interface, because only temporally adaptive systems can convert early adhesion into durable tissue repair and long‐term functional integration [32].

Together, these mechanisms indicate that the function of 3D‐printed bioadhesives lies not only in tissue bonding, but in shaping a proliferative niche through coordinated regulation of interfacial priming, mechanics, transport, signalling and temporal remodelling. The key question that follows is how these common principles are adapted across different repair settings. The following section therefore focuses on their application‐specific translation in wound management, musculoskeletal regeneration, wearable and implantable biointerfaces and organoid‐related models.

## 4 Application Landscapes: Tissue‐Specific 3D‐Printed Bioadhesives for Repair

3D‐printed bioadhesives are ultimately defined not only by how they adhere, but by how they perform in specific tissue settings. Because different repair contexts impose distinct demands on interfacial mechanics, transport, bioactivity and durability, application‐aware design is essential. This chapter therefore examines representative scenarios in which 3D‐printed bioadhesives may support proliferative‐niche formation across wound repair, musculoskeletal regeneration, bioelectronic interfacing and organoid‐related models. Where direct proliferation data are unavailable, the relevant systems are discussed as examples of interfacial design features that may enable proliferative‐niche formation rather than as established proliferative niches.

### 4.1 Wound Sealing and Haemostasis

Wound sealing and haemostasis are among the most clinically mature application settings for 3D‐printed bioadhesives, in which printing‐enabled architectures provide fluid‐resistant sealing, conformal defect matching and integrated pro‐healing functions beyond conventional cast or spray‐applied systems [6, 8]. Rather than merely shaping adhesives into predefined forms, 3D printing transforms them into programmable interfacial systems. Representative examples include adaptive PTLA hydrogel bioadhesives that achieve robust wet‐tissue adhesion and rapid ex vivo sealing on wet brain and lung tissues (Figure 4a–c) [8]. Direct‐ink‐write adhesive meshes with tunable geometries further improve conformability, handling and defect coverage, illustrating how printed architectures can refine interfacial contact and deployment in irregular wound settings (Figure 4d–f) [6]. Importantly, these strategies do not simply improve closure, but regulate blood exclusion, tissue contact and burst‐pressure performance under hemorrhagic conditions, thereby stabilizing the wound interface at the earliest stage of repair [6]. From a proliferative‐niche perspective, this early sealing effect is significant because it limits fluid leakage, contamination and mechanical disturbance, creating a more permissive microenvironment for subsequent cell survival and reparative progression. An emerging extension is in situ bioadhesive construction through imaging‐guided deep tissue sound printing (Figure 4g–j), which broadens the field from preformed patches towards anatomically localized adhesive fabrication [33].

**FIGURE 4:**
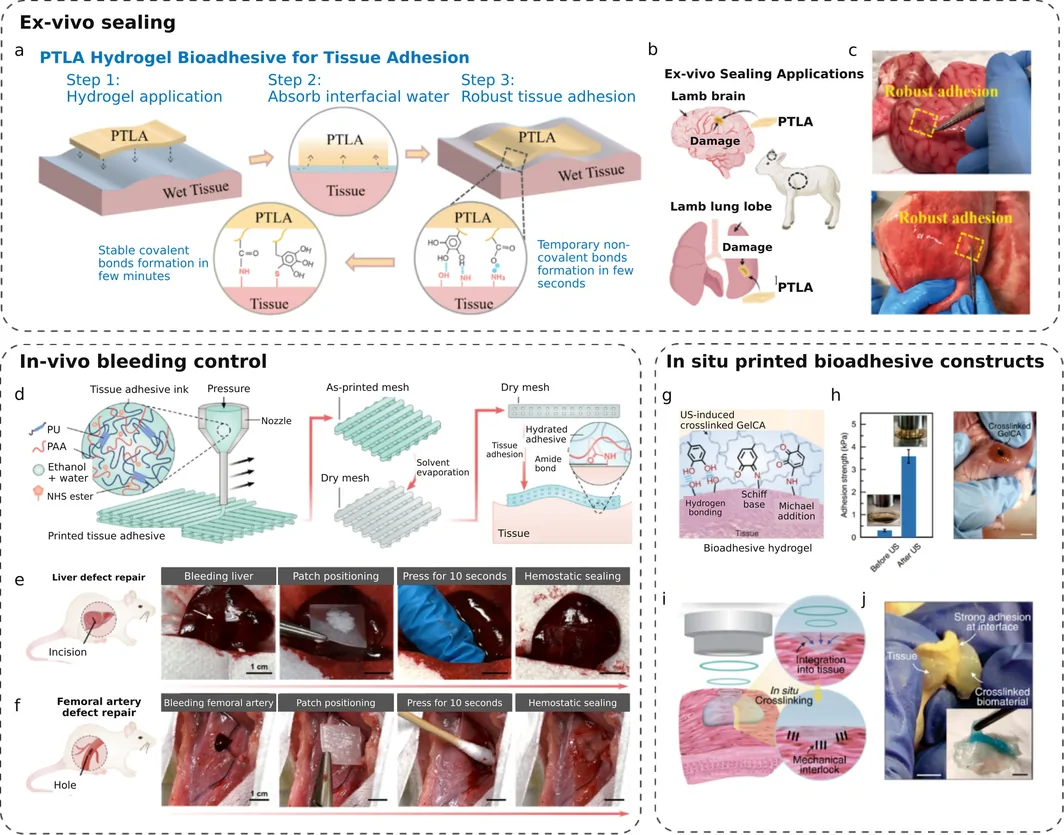
3D‐printed bioadhesives for ex vivo sealing, in vivo bleeding control and in situ bioadhesive construction. (a–c) Ex vivo sealing by a PTLA hydrogel bioadhesive, illustrating interfacial water absorption, rapid temporary interfacial interactions and robust adhesion on wet brain and lung tissues [8]. (d–f) In vivo bleeding control using a direct‐ink‐write 3D‐printed adhesive mesh patch, including blood‐resistant sealing of actively bleeding liver and femoral artery defects [6]. (g–j) In situ printed bioadhesive constructs enabled by imaging‐guided deep tissue ultrasound printing, showing ultrasound‐triggered crosslinking, enhanced adhesion, tissue integration and strong interfacial fixation [33].

### 4.2 Musculoskeletal Tissue Repair: Bone, Tendon and Cartilage

Musculoskeletal repair places unusually stringent demands on bioadhesives because successful regeneration requires more than wet fixation at the defect site; it also requires mechanical compliance, structural compatibility and sustained biological support within a dynamic load‐bearing environment. In this context, 3D printing is valuable not simply as a fabrication tool, but as a means to programme interfacial mechanics, spatial architecture and functional heterogeneity into a regenerative microenvironment. A representative starting point is robust interface coupling: the Velcro‐inspired PEGgel platform (Figure 5a,b) shows how tunable mechanics and strong hydrogel–device–tissue adhesion can stabilize complex musculoskeletal interfaces, helping shift bioadhesion from passive fixation towards an active basis for proliferative‐niche formation [12].

**FIGURE 5:**
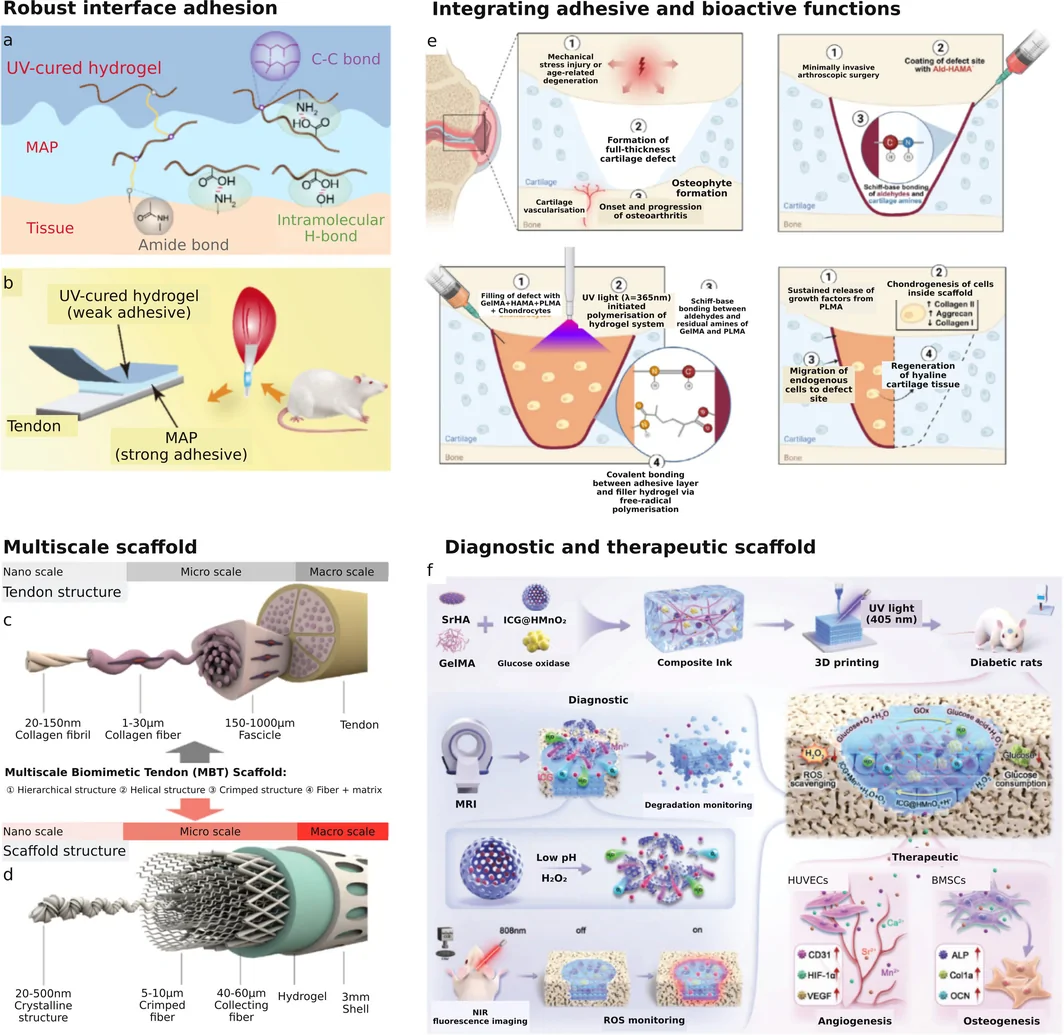
3D‐printed bioadhesive strategies for musculoskeletal tissue repair. (a, b) Robust interface adhesion is exemplified by a Velcro‐inspired PEGgel platform, in which tunable mechanics and strong interfacial bonding enable compliant coupling between hydrogel, device and tissue [12]. (c, d) A multiscale biomimetic tendon scaffold integrating a porous, sutureable PCL shell with GelMA‐containing crimped microfibers and nanohybrid shish‐kebab topography, thereby coupling mechanical transmission with tendon stem/progenitor‐cell adhesion and proliferation [13]. (e) Integrating adhesive and bioactive functions for cartilage regeneration is achieved through an injectable dual‐layer hydrogel, combining an adhesive primer with a bioactive overlayer for stable fixation and sustained chondrogenic support [53]. (f) A diagnostic and therapeutic scaffold for bone repair illustrates a bioadaptable 3D‐printed platform that couples microenvironment‐activatable visualization with osteogenic regeneration in diabetic bone defects [52].

Beyond fixation, effective tendon and cartilage repair depends on whether bioadhesive constructs can recreate tissue‐relevant structural and biological cues. For tendon regeneration, the multiscale biomimetic tendon (MBT) scaffold illustrates both the value and the limitations of applying the six‐part framework to a printed regenerative construct (Figure 5c,d). Interfacial fixation was achieved primarily through surgical suturing of the porous PCL shell rather than intrinsic wet‐tissue adhesion; thus, this system is more appropriately considered a boundary case for 3D‐printed bioadhesive repair. Mechanical and transport requirements were coupled through the shell architecture. A 45° printing angle was selected because the mechanically stronger 90° architecture produced larger fibers that were more difficult to degrade, whereas excessively compact architectures could restrict cell immersion and growth. The assembled scaffold reached a tensile strength of approximately 6.9 MPa and a modulus of 71.3 MPa, while its interconnected pores supported cellular and vascular infiltration. Biological instruction was provided by the RGD‐rich GelMA phase and crimped microfibers bearing a nanohybrid shish‐kebab topography, which enhanced tendon stem/progenitor‐cell adhesion and proliferation. Temporally, the PCL component provided prolonged mechanical support, with most of the scaffold degraded by 7 months in a rabbit patellar‐tendon defect model. Printability was achieved by combining rotational fused‐deposition modelling melt electrowriting, crystallization and 405‐nm photocrosslinking, although this multimodal workflow increases manufacturing and sterilization‐validation requirements. The system therefore trades intrinsic wet‐tissue adhesion and process simplicity for sutureability, mechanical support, transport‐permissive architecture and multiscale biological instruction [13, 34, 54]. For cartilage repair, the challenge shifts towards stable integration in a fluid‐filled joint together with sustained chondrogenic support. The injectable dual‐layer system (Figure 5e) addresses this by combining an adhesive Ald‐HAMA primer with a bioactive GelMA/HAMA/PLMA overlayer [53].

This trajectory becomes even clearer in bone repair, where scaffolds are evolving from structural supports into adaptive microenvironmental regulators. In diabetic bone defects, the bioadaptable 3D‐printed scaffold (Figure 5f) links microenvironment‐activatable visualization with osteogenic regeneration, extending musculoskeletal bioadhesives towards theranostic proliferative‐niche engineering [52]. Taken together, these studies show that 3D‐printed musculoskeletal bioadhesives function best when adhesion, architecture and biological instruction are integrated as a coordinated regenerative system [12, 13, 52, 53].

### 4.3 Bioelectronic Interfaces: Wearable and Implantable Systems

Bioelectronic interfaces place particularly stringent demands on 3D‐printed bioadhesives because these systems must maintain stable tissue contact while simultaneously supporting signal transmission, mechanical compliance and long‐term biological tolerance. In wearable settings, the challenge is to preserve conformal epidermal integration under continuous motion, sweat exposure and repeated deformation without introducing irritation, occlusion or signal instability [55, 56]. In this context, 3D printing is valuable not simply for device fabrication, but for enabling patterned architectures, user‐specific geometries and integrated multifunctionality that are difficult to realize through conventional assembly. A representative example is the Ag flakes–reinforced conductive bio‐adhesive platform, which combines strong adhesion, high conductivity and direct on‐skin patterning into a unified sensing interface (Figure 6a) [57]. A complementary direction is provided by DLP‐printed poloxamer micelle hydrogels, in which printable resin design and post‐print ionic conversion generate programmable wearable sensors with anti‐freezing, anti‐swelling and adaptive mechanical behaviour (Figure 6b) [58]. Together, these systems show that wearable bioadhesives are evolving from fixation materials into engineered epidermal interfaces that coordinate adhesion, softness, environmental adaptability and functional signal acquisition [57, 58, 59].

**FIGURE 6:**
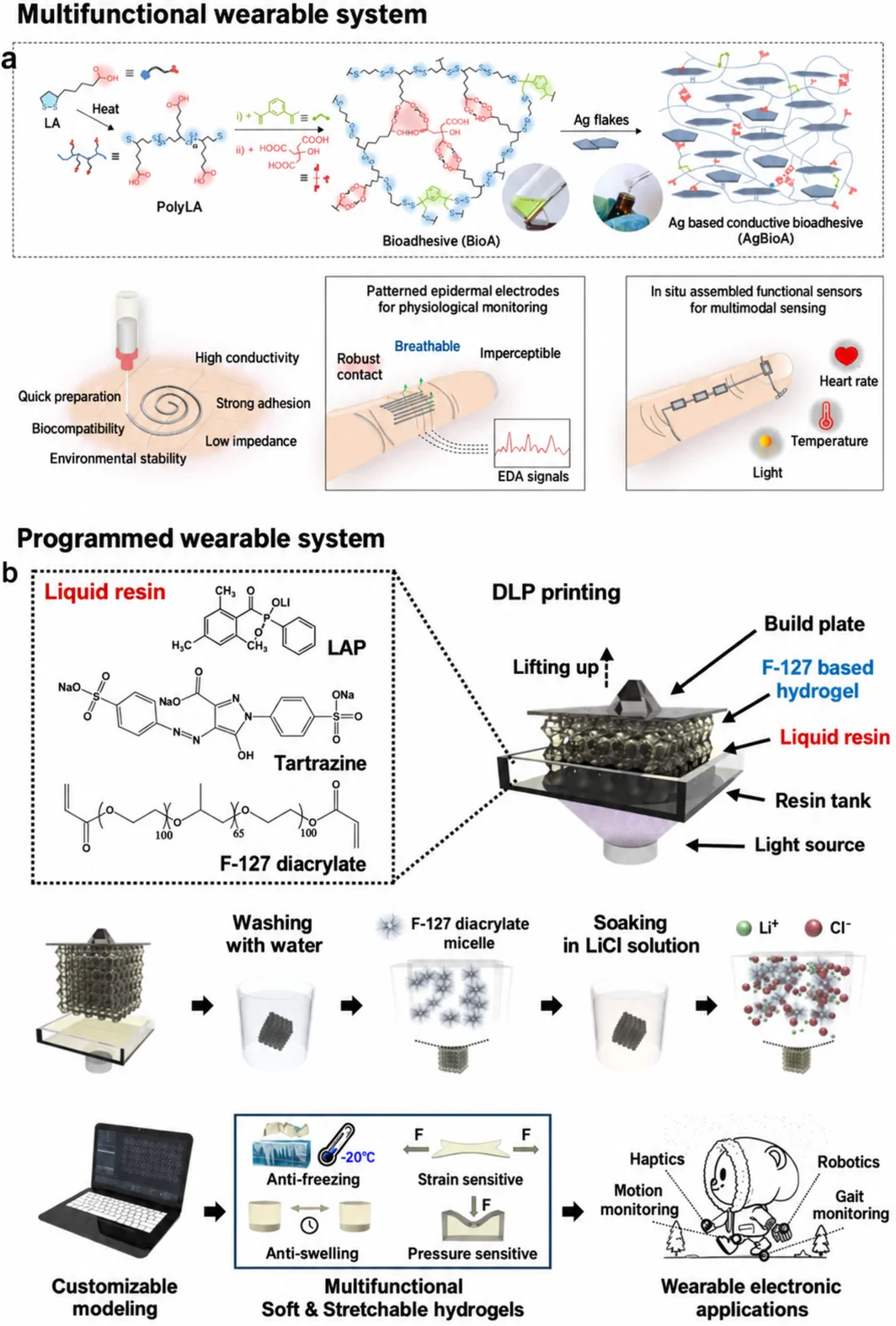
3D‐printed wearable bioadhesive systems for health monitoring. (a) A multifunctional wearable system based on Ag flakes‐reinforced conductive bio‐adhesive integrates material preparation, direct writing on skin, patterned epidermal electrodes and multimodal physiological sensing [57]. (b) A programmed wearable system based on DLP‐printed poloxamer micelle hydrogels illustrates printable resin design, post‐print ionic conversion and customizable soft sensors for motion and tactile monitoring [58].

In implantable settings, the design criteria become even more demanding because short‐term attachment is insufficient and long‐term functional integration defines performance. In the heart, for example, interfaces must accommodate cyclic contraction while maintaining stable sensing and stimulation and avoiding trauma during deployment or retrieval. The 3D‐printable bioadhesive pacing lead (Figure 7a) provides a representative example in which the six design dimensions were optimized primarily for functional bioelectronic integration rather than tissue regeneration. Polyacrylic‐acid‐grafted hydrophilic polyurethane and EDC/sulfo‐NHS chemistry generated physical and covalent bonding to epicardial tissue, whereas cleavable disulphide linkages and a bicarbonate/glutathione solution enabled on‐demand removal. This design illustrates the trade‐off between strong fixation and atraumatic retrievability. Mechanical matching was supported by swollen Young's moduli of approximately 90 kPa for the nonconductive bioadhesive and 490 kPa for the PEDOT conductive layer, both within the reported range of cardiac tissue, although the higher modulus of the conductive formulation reveals a conductivity‐compliance trade‐off. Anisotropic swelling was used to reduce geometric mismatch and a printed fluidic reservoir enabled transport of the detachment solution; however, nutrient permeability and longer‐term swelling‐induced tissue effects were not evaluated. Multimaterial direct‐ink writing integrated the electrode, insulation, bioadhesive interface and fluidic reservoir, but also increased the burden of scalable and reproducible manufacturing. Function and tissue compatibility were maintained for up to 2 weeks in rodent and porcine models, yet the study did not include a cell‐proliferation endpoint. Accordingly, this interface should be interpreted as niche‐compatible rather than as a demonstrated proliferative niche. Its long‐term adhesion, electrical stability, fibrotic response and industrial manufacturing reproducibility remain unresolved translational questions [20]. In the nervous system, the challenge shifts towards whether the implanted construct can provide a structurally instructive microenvironment for regeneration. The NEAT bioprinting strategy addresses this by generating anisotropic neural constructs that mimic spinal cord extracellular matrix architecture and guide hNSC alignment and neuronal differentiation (Figure 7b) [42].

**FIGURE 7:**
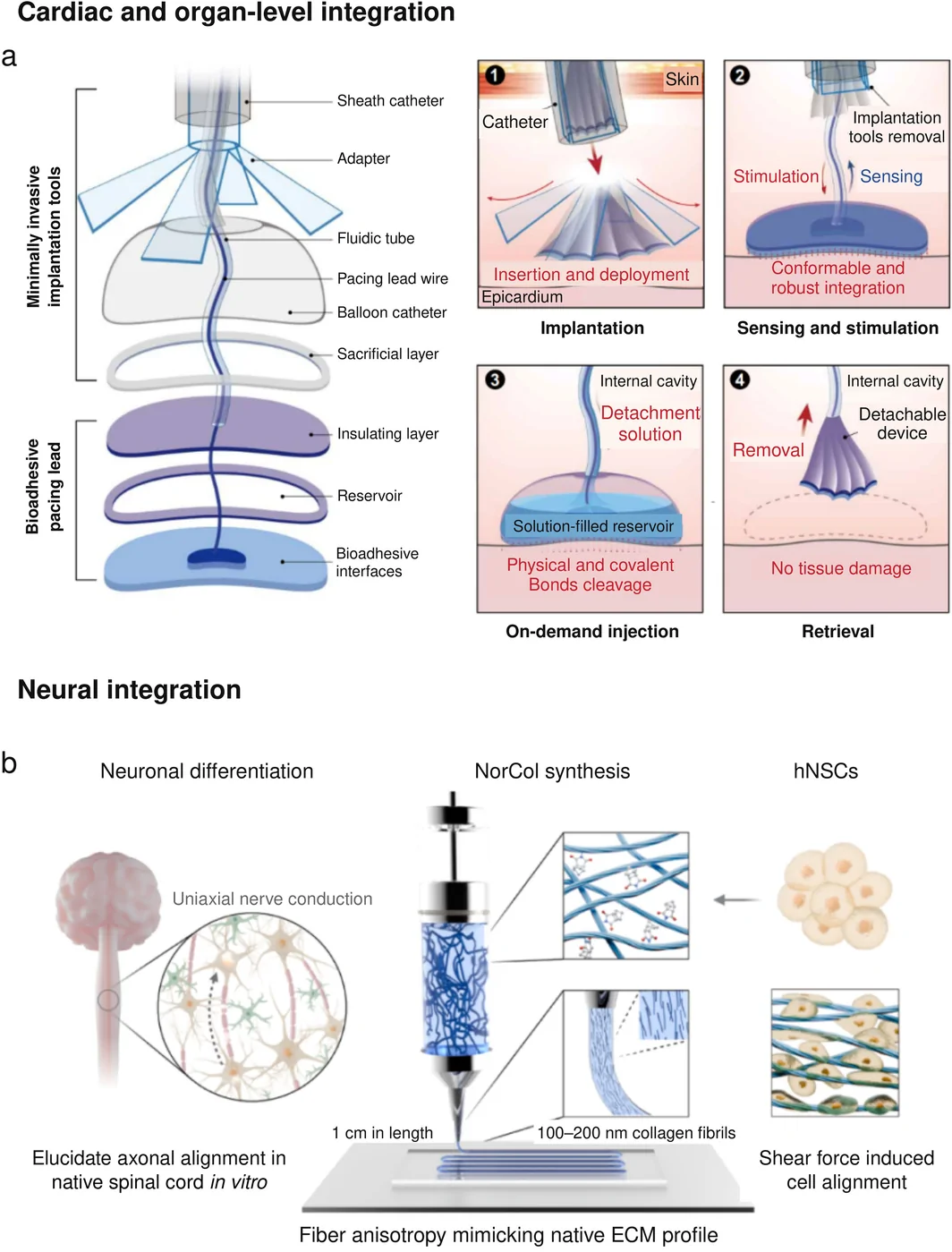
Implantable printed bioadhesive interfaces for cardiac, organ‐level and neural integration. (a) A 3D‐printable bioadhesive pacing lead illustrates minimally invasive deployment, conformal epicardial integration for sensing and stimulation, solution‐triggered debonding and atraumatic retrieval, highlighting cardiac and organ‐level bioelectronic interfacing [20]. (b) Nanoengineered extrusion‐aligned tract bioprinting generates anisotropic neural constructs that mimic spinal cord extracellular matrix architecture and guide hNSC alignment and neuronal differentiation, highlighting structure‐directed neural integration after implantation [42].

Taken together, these examples illustrate how selected bioelectronic interfaces can provide combinations of adhesion, mechanics, transport and electrical or structural bioinstruction that may enable proliferative‐niche formation. Whether a given interface qualifies as a proliferative niche further depends on measurable, context‐appropriate biological responses. In wearables, the dominant goal is stable long‐term epidermal coexistence; in implantables, it extends towards durable organ‐level integration and regeneration‐guiding interfacing over time [20, 42]. Accordingly, tissue compatibility, electrical integration and stable device function should be regarded as niche‐enabling attributes unless context‐appropriate proliferation or remodelling outcomes are directly demonstrated.

### 4.4 Engineered Biointerfaces for Organoid Models

Engineered biointerfaces for organoid models are important because bioprinting does not merely define construct shape, but establishes the interfacial conditions under which self‐organization, proliferation, lineage progression and tissue patterning can occur. As summarized in Figure 8, relevant design variables include cell density, shear exposure during deposition, remodellable matrix properties, organoid surface integrity, stiffness, yield stress, shear‐thinning behaviour, spatial positioning and transport constraints [60]. This interface‐centred control is particularly valuable because many conventional organoid‐culture protocols rely on poorly defined matrices and minimally regulated self‐assembly, which can contribute to heterogeneity in size, morphology and developmental state [60, 61].

**FIGURE 8:**
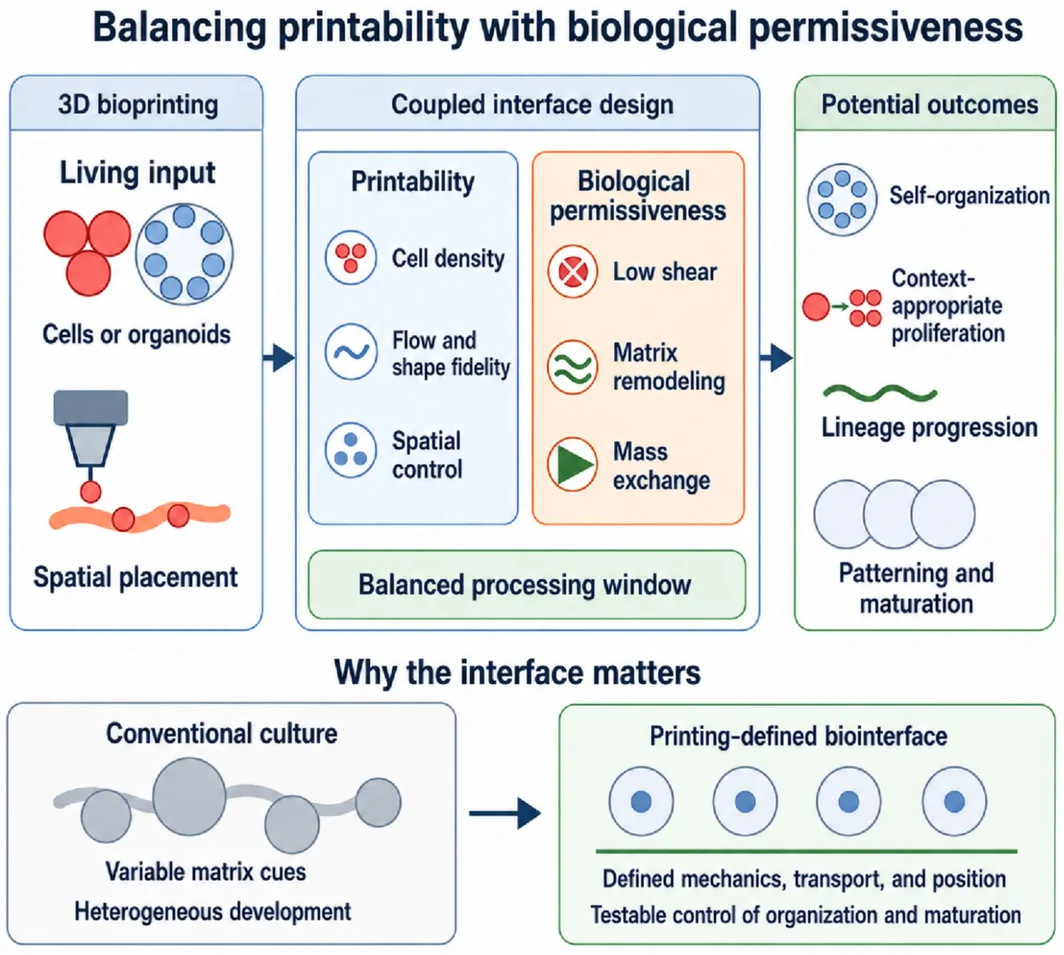
Coupling printability with biological permissiveness in engineered organoid biointerfaces. Bioprinting coordinates cell density, stiffness, yield stress, shear‐thinning behaviour and spatial positioning with limited shear exposure, preserved organoid surface integrity, matrix remodellability and stress relaxation and mass exchange. These matched features may support self‐organization, context‐appropriate proliferation, lineage progression, tissue patterning and maturation, subject to direct biological validation. In contrast, poorly defined matrices and minimally regulated self‐assembly contribute to heterogeneity in size, morphology and developmental state [19, 60, 61, 62].

From a proliferative‐niche perspective, the key requirement is to couple printability with biological permissiveness. Matrices must protect organoid‐forming cells from deposition‐induced damage while remaining sufficiently dynamic to support post‐print self‐organization, controlled fusion and mass exchange [60]. In this setting, the target population comprises the defined printed or embedded organoid‐forming cells rather than the organoid itself as a cell type; their proliferation should be interpreted together with lineage progression and tissue organization rather than maximized in isolation. Accordingly, rheology and viscoelasticity become biologically consequential rather than purely processing‐related variables. High‐density or jammed suspensions improve placement precision, whereas yield‐stress, shear‐thinning, stress‐relaxing and dynamically tunable matrices better support morphogenesis and maturation across organoid systems [19, 60, 61, 62]. Thus, engineered organoid biointerfaces can serve as controlled in vitro testbeds for proliferative‐niche design rather than as established tissue‐repair proliferative niches; their predictive relevance to host‐tissue bioadhesive interfaces requires direct validation of application‐relevant adhesion and context‐appropriate biological outcomes [60, 61].

### 4.5 Application‐Specific Design Trade‐Offs and Common Failure Modes

Across tissue applications, the performance of 3D‐printed bioadhesives is defined less by any single optimal property than by how effectively competing requirements are balanced within a given biological context [1, 7]. Adhesion strength, degradation kinetics, permeability, printability, mechanical compliance and multifunctionality rarely improve in parallel, meaning that successful designs are inherently application‐specific rather than universally ideal [1, 54]. From a proliferative‐niche perspective, this is important because interfacial features that stabilize repair in one setting may impair biological permissiveness in another. For example, strong fixation may be beneficial for sealing wet or bleeding tissues, yet excessive crosslinking or stiffness can suppress interpenetration, stress relaxation and constructive remodelling [7]. Likewise, porous or mesh‐like architectures may improve transport and fluid handling while compromising cohesion or mechanical uniformity [6].

Accordingly, the most common failure modes are best understood as unresolved trade‐offs rather than isolated material defects. Fabrication‐associated failures include spreading, nozzle clogging, incomplete curing, loss of geometric fidelity and process‐induced biological damage [6, 34, 54]. In cell‐laden extrusion, pre‐crosslinking can improve structural fidelity while increasing viscosity and the shear stress imposed on encapsulated cells; screw‐driven extrusion has also been reported to cause greater cell damage than pneumatic printing [54]. For photocurable systems, increasing photoinitiator concentration can accelerate curing and improve fidelity, but excessive radical generation can be cytotoxic, while inadequate washing or post‐curing may leave unreacted species within the construct [34]. Nonuniform light penetration can additionally cause under‐curing, over‐curing, mechanical heterogeneity or interlayer delamination [34]. Interfacial and temporal failures include weak wet contact, leakage, motion‐induced delamination, swelling‐induced tissue mismatch, premature degradation and insufficient fracture resistance [6, 20, 63]. In multifunctional systems, added conductivity, removability or responsiveness may introduce further vulnerabilities. Print fidelity should therefore be evaluated together with post‐print cell viability and proliferation, residual‐species analysis, cyclic interfacial stability and application‐specific functional performance [34, 54].

Taken together, these application contexts show that the value of 3D‐printed bioadhesives lies not in maximizing any single property, but in balancing adhesion, mechanics, transport and biological instruction in a tissue‐specific manner. This also explains why strong short‐term performance does not necessarily predict long‐term proliferative‐niche function. A more rigorous framework is, therefore, needed to evaluate how printed bioadhesive interfaces support proliferation, integration and remodelling over time, motivating the multidimensional assessment and predictive modelling strategies discussed in the next section.

### 4.6 Clinical Translation Bottlenecks

The representative systems discussed above remain at the preclinical stage. The bioadhesive pacing lead was evaluated in rodent and porcine models for up to 2 weeks. The MBT tendon scaffold was studied in a rabbit tendon‐defect model for up to 28 weeks, with additional observations at 7 months. Neither primary report described human implantation [13, 20]. More broadly, a 2025 review identified only four ClinicalTrials.gov records using the term ‘3D bioprinted’, none corresponding to these bioadhesive platforms [54]. This time‐specific observation illustrates the limited clinical exploration of the field but should not be interpreted as establishing the absolute absence of ongoing trials. Regulatory classification will depend on cellular content, primary mode of action and the degree of functional integration. Acellular constructs may follow medical‐device pathways, whereas cell‐containing or functionally complex constructs may require Class III, biologic, advanced‐therapy or combination‐product review [54]. Personalized geometry and multimaterial construction additionally require validated CAD workflows, material controls, printer qualification, process validation, manufacturing tolerances, lot‐release criteria and traceable quality assurance [34, 54].

Clinical failure may occur even when short‐term adhesion is successful. Relevant risks include swelling‐induced geometric mismatch or tissue irritation, cyclic fatigue and delamination under dynamic loading, degradation that is mismatched to tissue healing, fibrosis, residual reactive species and loss of electrical or mechanical function [20, 34]. The pacing‐lead study used anisotropic swelling to reduce mismatch and reported stable performance for 14 days, but the authors identified longer‐term adhesion, electrical performance, fibrosis and scalable manufacturing as unresolved issues [20]. Its preclinical preparation involved aseptic handling followed by 1 h of UV exposure, which does not by itself establish compatibility with a clinically validated terminal‐sterilization process. The tendon study likewise did not report a clinically validated sterilization workflow in the main article. Clinical implementation will therefore require evaluation of sterilization effects on adhesion, conductivity, degradation, bioactivity and residual cytotoxicity, together with shelf‐life, packaging, repeated wet‐fatigue testing and longer‐term large‐animal studies [34, 54].

## 5 Evaluation and Modelling Frameworks for Proliferative‐Niche Engineering

Evaluating 3D‐printed bioadhesives through a proliferative‐niche lens requires moving beyond single metrics such as adhesion strength or short‐term cytocompatibility towards integrated frameworks that relate interface structure, material properties, biological responses and functional outcomes. As illustrated in Figure 9, this chapter examines how multidimensional assessment, cross‐scale structure–property–function mapping and data‐driven modelling can be used to determine whether a bioadhesive interface is not only printable and adhesive, but also capable of sustaining cell proliferation, constructive remodelling and durable tissue integration. Evaluation, in this sense, is not merely descriptive, but central to building an integrated understanding of proliferative‐niche performance.

**FIGURE 9:**
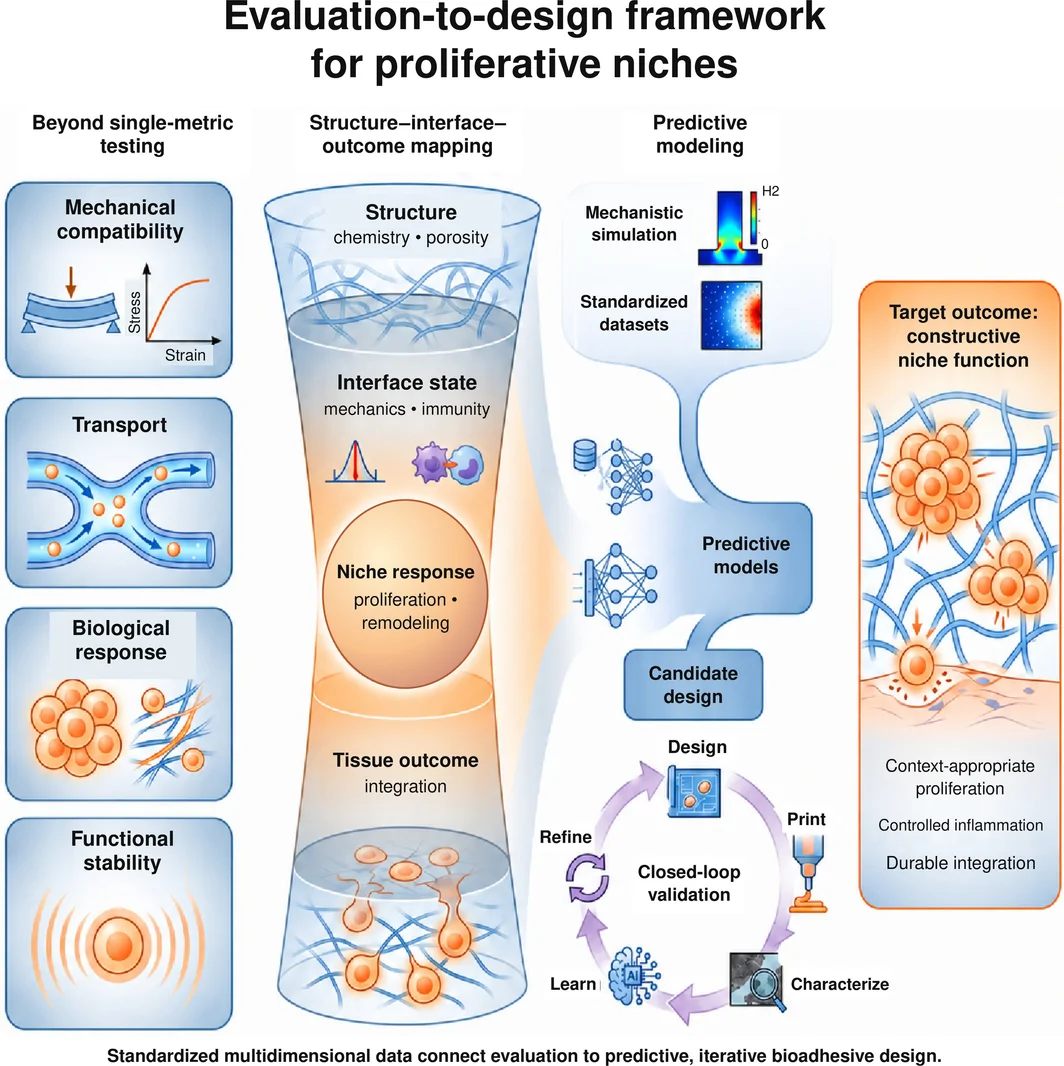
Evaluation and modelling framework for proliferative‐niche engineering in 3D‐printed bioadhesives. Multidimensional interfacial assessment, cross‐scale mapping and physics‐informed/data‐driven modelling converge towards predictive and eventually adaptive optimization of proliferative niches at bioadhesive interfaces for improved proliferation, reduced inflammation and durable tissue integration.

### 5.1 Beyond Adhesion Strength: Multidimensional Evaluation of Interface Performance

Standard adhesion tests remain necessary, but they capture only one dimension of interface performance and therefore cannot determine whether a 3D‐printed bioadhesive truly establishes a constructive proliferative niche. For interfaces intended to support tissue remodelling or long‐term bioelectronic integration, evaluation should move beyond single‐value bonding metrics. It should include interfacial mechanics, transport behaviour biological compatibility and application‐specific functional stability. A material may adhere strongly during initial testing but later fail because of swelling, fatigue, signal instability, inflammatory intolerance or loss of conformal contact [1, 16, 54].

### 5.2 Biological Readouts for Proliferative‐Niche Assessment

Biological evaluation should move beyond simple viability and cytotoxicity testing, because transient cell tolerance does not necessarily indicate that a 3D‐printed bioadhesive has established a proliferative niche [4, 26]. A more meaningful framework should, therefore, combine proliferation‐associated readouts that distinguish survival from active regenerative participation [4, 64], inflammatory‐state assessment [26, 29] and matrix‐remodelling and tissue‐integration trajectories across acute and longer‐term windows. Where feasible, proliferation should be evaluated according to the target‐cell population and repair stage and interpreted against tissue‐specific repair goals rather than assuming that greater proliferation is uniformly beneficial. Only these combined outcomes can distinguish short‐lived compatibility from sustained proliferative‐niche function [26, 64].

### 5.3 Structure–Property–Function Correlations Across Scales

A central challenge is to move beyond isolated descriptors and determine how material chemistry, crosslinking state and micro−/meso‐scale architecture are translated into emergent biological and functional outcomes over time [7, 10]. In 3D‐printed bioadhesives, this requires correlation frameworks that connect structural variables such as network organization, stiffness, viscoelasticity, pore or pattern geometry and hierarchical anisotropy with downstream effects on cell behaviour transport, tissue integration and interface durability [13, 19]. Such cross‐scale mapping is essential because many measured variables merely co‐vary, whereas only a smaller subset is truly predictive of proliferative‐niche function and application‐level performance [7, 65].

### 5.4 Data‐Driven Modelling, Predictive Design and Closed‐Loop Optimization

Data‐driven modelling is increasingly needed because empirical trial‐and‐error screening alone is too slow and too fragmented to navigate the coupled design space of 3D‐printed bioadhesive interfaces [54, 66]. Predictive frameworks can help prioritize formulations, structures and process windows by linking print parameters, material descriptors and interfacial measurements to expected outcomes such as fidelity, stability and biological performance relevant to proliferative‐niche formation [54, 65, 67]. However, their broader usefulness depends on standardized datasets, biologically meaningful labels and careful treatment of cross‐study heterogeneity; otherwise, models risk becoming workflow‐specific fitting tools rather than transferable design engines [54, 66, 67]. The next step is to connect predictive modelling with iterative experimentation, real‐time monitoring and parameter updating within the fabrication workflow, so that modelling, printing, characterization and retraining are coupled in a closed‐loop manner [66, 67]. In this framework, the goal is not only to improve print fidelity and process robustness, but also to converge more efficiently towards biologically favourable proliferative‐niche states across engineering and biological readouts [54, 65].

Current evaluation and modelling frameworks have moved the field beyond one‐dimensional descriptions of adhesion, biocompatibility or printability towards a more integrated understanding of proliferative‐niche performance. Yet most existing approaches still provide only partial predictive control over interface formation and remodelling across time. This limitation points naturally to the next question: how adaptive, data‐linked and clinically relevant platforms may be built to guide future proliferative‐niche engineering.

## 6 Future Directions and Outlook: Towards Adaptive and Predictive Proliferative‐Niche Engineering

Self‐driving bioprinting laboratories, closed‐loop fabrication frameworks and related digital‐twin concepts represent a promising direction for proliferative‐niche engineering because they offer a way to connect automated fabrication, multimodal characterization and model updating across engineering and biological timescales [54, 65, 67]. In this context, the key opportunity is not simply to optimize print fidelity or throughput, but to iteratively improve outcomes relevant to proliferative‐niche formation such as curing behaviour, swelling control, cell retention, inflammatory tone and remodelling quality. Over time, such adaptive systems may allow 3D‐printed bioadhesives to be designed not only as predefined materials, but as dynamically learnable interfaces whose composition, architecture and processing conditions are progressively tuned towards more robust proliferative‐niche formation. Infected bone defects provide a representative application context for this convergence: recent reviews emphasize the need to coordinate 3D‐printing‐enabled structural control, hydrogel‐mediated immune‐microenvironment regulation, antimicrobial function and osteogenic regeneration, while AI‐assisted and multidimensional printing may expand the available design space [68, 69, 70].

At the same time, translational progress will depend on whether these concepts can be reconciled with the practical constraints of clinical manufacturing and regulation [7, 54]. Future systems must therefore address issues such as reproducible scale‐up, sterilization compatibility, shelf stability, multimaterial process robustness and standardized biological validation, while also fitting evolving regulatory expectations for combination products, printed biomaterials and adaptive manufacturing workflows. In this sense, the future of 3D‐printed bioadhesives will depend not only on smarter design tools, but on whether adaptive proliferative‐niche engineering can be translated into manufacturable, testable and clinically actionable platforms for tissue repair.

## Author Contributions

Z.G., Y.W. and Q.G. designed the review and made a retrieval strategy. Z.G. and X.S. drafted the review text. Z.G. and G.X. drafted the tables and figures. Z.G., S.X., G.X., Y.W. and Q.G. contributed to the revision and finalization of the manuscript.

## Funding

This work was supported by the National Key Research and Development Program of China (2024YFB4607800, 2025YFC3408800), National Natural Science Foundation of China (52273120), Initiative Scientific Research Program of Institute of Zoology (202310Z0101, 2025IOZ02) and the China Scholarship Council (202206465017).

## Conflicts of Interest

The authors declare no conflicts of interest.

## Data Availability

Data sharing not applicable to this article as no datasets were generated or analysed during the current study.
